# An investigation into how accurately UK rabbit owners identify pain in their pet rabbits

**DOI:** 10.1186/s12917-024-03947-7

**Published:** 2024-03-27

**Authors:** Charlotte Forder, Livia Benato, Nicola J. Rooney

**Affiliations:** Animal Welfare and Behaviour Group, Bristol Veterinary School, University of Bristol, Langford, BS40 5DU UK

**Keywords:** Pet rabbit, Pain score, Demographics, Owner ability

## Abstract

**Background:**

Rabbits are popular family pets. They are prey species and so have evolved to hide signs of illness and pain. Recent research has developed robust pain scales for use in a clinical setting, but to date none has examined rabbit owners’ ability to recognise pain in their animals. This study investigated how owners identify pain in their pet rabbits and their ability to correctly identify different levels of pain, in order to determine any need for owner education in this area.

**Methods:**

Owners were recruited via Facebook and a two-part online survey was distributed. Part one collected data on demographics, owners’ knowledge of pain signs and beliefs about pain in rabbits. Part two asked respondents to pain score eight videos of rabbits in different levels of pain for comparison to pain scores made by three experts. We used a simplified version of the Bristol Rabbit Pain Score (BRPS) which involved a single 0 to 3 scale. We explored the number of pain signs each respondent could list, the total score given to the videos, and their deviation from the experts’ scores.

**Results:**

A total of 500 respondents completed part one of the survey and 345 additionally completed Part two. Respondents were on average able to state five signs of pain (range 0–12), but females stated significantly more (*p* = 0.018), as did those who worked with rabbits (*p* = 0.004) and those with experience of their rabbit having an operation (*p* = 0.01). Overall, 98.6% of respondents thought rabbits felt pain as much or more that dogs and cats. In Part two, respondents more frequently agreed with the experts when identifying rabbits in no pain (88.8%) and severe pain (65.2%), but there was lower agreement when identifying mild (28.4%) and moderate pain (43.2%). Respondents overall rated pain lower than experts with an average total pain score of 11.9 compared to 18 given by the experts.

**Conclusions:**

Most rabbit owners are able to list numerous pain signs and are generally able to identify pain-free rabbits and those in severe pain. Owners’ ability to differentiate between mild and moderate pain is more limited and could benefit from training in the subtler signs of pain. Veterinary professionals are well placed to educate owners about signs of pain in rabbits and should be aware of areas where owners’ knowledge can be improved.

**Supplementary Information:**

The online version contains supplementary material available at 10.1186/s12917-024-03947-7.

## Background

Pain is described by the International Association for the Study of Pain (IASP) as an “unpleasant sensory and emotional response associated with, or resembling that associated with, actual or potential tissue damage” [1]. Furthermore, the World Small Animal Veterinary Association (WSAVA) states that pain is an experience that is perceived by all mammals and, as such, rabbits’ experience pain as do dogs and cats [2].

There are currently estimated to be a million pet rabbits in the UK, making them the third most popular mammalian pet behind cats and dogs [3]. Rabbit pain can be caused by a variety of reasons including disease, accidental injury and planned surgical procedures. A study investigating levels of pain associated with various surgical procedures, found that veterinarians rated orthopaedic procedures as more painful than neutering, surgical treatment of abscesses and tumour removal [4]. Other studies have found that conditions commonly occurring in pet rabbits such as gastrointestinal disease, fight wounds, urinary disease and neutering are all believed to cause significant pain [5]. These findings, alongside recent reports that 63% of rabbits are neutered [6] mean that most, if not all, pet rabbits will experience some level of pain during their lives. Most companion animals such as cats and dogs are predators, whereas rabbits are prey species [7, 8] and therefore have a high drive and ability to hide signs of illness and pain. Changes due to pain are often subtle, making them difficult to notice and they may seem insignificant to owners and carers within domestic settings [8].

Pain can be assessed by the physiological and behavioural changes it causes [9], but these can also be affected by environmental factors, the age and individual temperament of the rabbit. For example, heart rate, respiratory rate and temperature are useful physiological measures, but are also influenced by infection, stress and positive arousal [10]. Therefore, behavioural changes such as reduced appetite, faecal output, and exploratory movement are generally considered more reliable [11, 12].

Pain scales specific to rabbits have recently been developed, to allow assessment within a variety of settings. Bristol Rabbit Pain Scale (BRPS; [13–15]) is a tool available for veterinary professionals to identify and treat pain in rabbits in their care. It is based on behavioural indicators identified through a series of studies, as the most reliable indicators of pain [15]. It was validated using videos of rabbits before and after surgical procedures such as ovariohysterectomy and orchiectomy, and the cut-off point for intervention analgesia empirically determined [15]. This scale is designed to quantify acute pain in hospitalised patients primarily after surgery, to help veterinary professionals identify and treat pain.

Pain is commonly assessed and treated while rabbits are under veterinary care [16, 17]). However, as per the Animal Welfare Act of 2006 [18], it is also an owner’s responsibility to ensure that animals within their care do not suffer unnecessarily. This includes keeping them free from pain (as stipulated in the five welfare needs) by seeking prompt veterinary advice when necessary. Thus, it is imperative that owners are able to spot signs of pain, to ensure appropriate and timely veterinary interventions are sought and suffering is minimised. Despite this requirement, currently there is no research into how owners identify and assess pain in their rabbits. Moreover, the current pain assessment tools have been developed primarily to quantify acute, post-operative pain and not for use by owners. Therefore, it would be useful to know how rabbit owners currently identify pain at home and whether they are able to accurately identify different levels of pain. Education of owners is often cited as integral to protecting animal welfare, but it is vital to understand current levels of knowledge and common misconceptions, to optimally target future educational initiatives.

The current study aims to gain an understanding of the baseline level of knowledge of rabbit owners in the United Kingdom (UK) surrounding pain recognition in their pet rabbits. There is currently no research into factors that might influence owners’ assessment of pain in their rabbits or other species. This study hopes to gain understanding of this and potentially highlight areas of misunderstanding so that educational initiatives can be targeted to maximally benefit animal welfare. We explored whether owners are able to assess pain accurately using a four-point pain scale modified from Benato et al. [13] where 0 describes a rabbit in no pain, 1; mild pain, 2: moderate pain and 3: extreme pain. We also investigated whether there are factors that influence owners’ knowledge and ability to accurately assess pain. We hypothesised that length of ownership, as well as previous exposure to rabbits undergoing surgery may have an impact.

## Results

A total of 500 responses were received. The majority were female (94.8%), most often aged 25–34 years (29.0%; Table 1). Respondents had most commonly been a rabbit owner for 6–10 years (27.4%) and currently owned two rabbits (38.6%). A small percentage (11.4%) did not currently own a rabbit, with the time since owning a rabbit ranging from less than a year to 21 years (mean = 4.4 years). A large proportion of participants had five or more rabbits, previous to those they currently owned (40.6%), but 13.6% were first time owners.

**Table 1:** Demographic information about the 500 participants

Demographic variable	Percentage of respondents (%)
Gender	
Male	4.6
Female	94.8
Gender variant / non-conforming	0.6
Age Group	
16–24 years	11.6
25–34 years	29.0
35–44 years	22.0
45–54 years	22.0
55–64 years	12.8
65–74 years	2.4
75 +	0.2
Worked with rabbits professionally	
No	81.0
Veterinary surgeon (VS)	1.0
Veterinary nurse (VN)	4.4
Animal carer	5.2
Pet outlet	2.4
Rehoming centre	3.6
Veterinary role (not VS or VN)	1.6
Other	0.8
Time been a rabbit owner	
Less than 6 months	1.0
6 months up to one year	4.0
1–5 years	24.2
6–10 years	27.4
11–15 years	17.8
16 years +	25.6
Current number of rabbits owned	
0	11.4
1	22.0
2	38.6
3	8.4
4	8.6
5 +	11.0
Number of rabbits owned previously	
0	13.6
1	12.0
2	13.6
3	10.4
4	9.8
5 +	40.6
Caregiver	
Primary caregiver	76.6
Myself and another adult in the household	19.2
Myself and a child/children in the household	2.0
Another adult within the household	1.4
Child/Children in the household	0.6
Living space for rabbit	
House rabbit with access to one or more rooms	49.4
Hutch or cage in the house without an attached run	2.4
Hutch or cage in a shed, garage or outbuilding without attached run	1.0
Hutch or cage in the garden without attached run	4.6
Hutch or cage in the house with an attached run	5.4
Hutch or cage in a shed, garage or outbuilding with attached run	4.2
Hutch or cage in the garden with attached run	10.6
A shed or other outbuilding in which they could freely roam	14.2
Other	8.2

Overall, 19% of participants had worked with rabbits as part of their profession most often as an animal carer (5.2%), or veterinary nurse (4.4%) or in a pet outlet (2.4%), whilst the remaining 0.8% had other professions. The majority of respondents had experienced a rabbit in their care having an operation (85.2%). Most reported experiencing one (31.2%) or two (33.4%) types of operation, however, this ranged up to five (1.2%). The most common operation experienced was neutering (78%), followed by dental surgery (46.4%). The other types were less common: 4.4% orthopaedic surgery, 1.4% foreign body removal surgery, 16.8% lump removal surgery, 11.6% other major surgery and 6.6% other minor surgery.

Respondents most commonly reported being able to observe what their rabbit(s) was doing for more than 6–12 h (29.3%). Similar percentages of respondents reported being able to observe for more than two but less than six hours (28.1%) or for twelve hours or more (25.9%), whilst few reported < 10 min (0.6%) or 10–29 min (3.0%). The majority were the rabbit(s)’ primary caregiver (76.6%), and most rabbits lived in the owner’s house (57.2%).

### Respondents’ beliefs about rabbit pain

The vast majority of respondents thought that rabbits felt pain to the same extent as other animals (91.8%). A further 7.8% thought that rabbits felt pain more than other animals and the remaining 0.4% were unsure.

Respondents reported learning about signs of pain from a variety of sources, with their own experience being the most common way (78.4%). A further 49% reported to have learnt from veterinary professionals, 33.8% from social media, 21% from books and 11.4% from friends and family. Other sources described by respondents included via rescue/ rehoming (2.2%), informal education such as webinars and the RWAF website and magazine (2.8%) and formal education such as university or college courses (2.8%).

### Signs of pain

Thirteen different pain signs were mentioned by respondents and the total number of pain signs described by individual respondents varied from zero to twelve, with a median of 5 (25th percentile = 3, 75th percentile = 6).

Considering the six signs that appear in the BRPS, respondents listed between 0 and 5 of these with the median being 2 (1, 3). The most commonly listed was a change in posture and the most commonly missed was a decrease in grooming behaviour.

The most commonly reported sign of pain was anorexia (83.6%), whilst the most common category mentioned from the BRPS [13] was posture (63.4%); the terms ‘hunched’ and ‘abdomen pressing’ were specifically cited by 46.4% and 13% of respondents respectively. Use of the Grimace scale as a way to identify pain was specifically mentioned by 4.8% of respondents (Table 2).

**Table 2:** Percentage of 500 respondents that mentioned different signs of pain including the six categories within the BRPS. Bold denotes the six BRPS (Bristol Rabbit Pain Scale) categories

Listed sign of pain	Percentage of respondents mentioning	Percentage reporting as the most important
Anorexia	83.6	47.4
**Demeanour**	62.4	20.8
Posture	63.4	12.6
Hunched^a^	46.4	
Abdomen pressing^a^	13.0	
Locomotion	44.4	3.8
Lethargy^a^	15.4	
**Change in behaviour**	28.6	
**Ear position**	13.4	
**Eye position**	13.0	0.8
**Decrease in grooming**	6.2	0.2
Teeth grinding	35.0	4.8
Grimace scale	4.8	0.6
Noises	9.6	1.2
Faecal changes	19.8	1.2
Physiological	10.6	
All		4.2
Other		1.2

^a^denotes terms from within the BRPS categories that were commonly mentioned by participants

Respondents commonly reported anorexia (47.4%) as the most important pain sign. This was followed by demeanour (20.8%) and posture (12.6%) with a further 4.2% stating that all pain signs were equally important when assessing pain (Table 3).

**Table 3:** Percentage of 500 respondents reporting different levels of pain for each condition and time frame selected to seek veterinary attention following detection of condition. Empty cells were not asked for the specific question

	Percentage of respondents reporting level of pain associated with the conditon				Percentage of respondents reporting attention needed within different timescales					
	Not painful	Somewhat painful	Painful	Severely Painful	Does not need veterinary attention	After 14 days	Within 8–14 days	Within 4–7 day	Within 2–3 days	Same day
Bone fracture	0.0	1.0	7.4	91.6	0.0	0.0	0.0	0.4	0.8	98.8
Overgrown back teeth	0.0	2.6	38.6	58.8	0.0	0.0	0.8	4.6	37.8	56.8
Gut stasis	0.2	3.2	23.8	72.8	0.0	0.0	0.2	0.2	2.8	96.8
Fly strike	0.6	4.8	19.0	75.6	0.4	0.0	0.0	0.6	4.2	94.8
Hock sores	0.0	8.0	46	45.2	1.4	0.4	0.8	6.8	42.0	48.6
Ear infection	0.2	6.6	42.8	50.4	0.2	0.0	0.0	2.6	28.8	68.4
Urine scalding	0.2	5.4	43.2	51.2	0.8	0.2	0.4	4.0	34.2	60.4
Neutering -female	2.4	20.0	44.6	33.0						
Neutering-male	2.8	29.4	45.8	22.0						
Hay poke in eye	0.4	17.2	47.6	34.8	3.6	0.6	0.4	1.2	18.4	75.8
Thorn in foot	0.2	16.0	51.6	32.2						
Bite wound	0.0	13.8	48.2	38.0	3.2	0.2	0.4	2.4	15.4	78.4
Osteoarthritis	0.0	12.0	56.0	32.0	0.6	2.2	3.6	17.8	48.6	27.2
Lump or swelling	1.6	35.2	47.8	15.4	0.4	0.6	2.2	10.4	39.2	47.2

The total number of pain signs listed by females (5 (3,6)) was significantly greater than by males (4 (2,5); MWU = 3882, *p* = 0.018), however age showed no significant correlation with total number of pain signs (Rho = -0.012, *p* = 0.795).

Respondents working with rabbits listed significantly more signs 5 (4,6) than those that did not (4 (3,6); MWU = 15,628, *p* = 0.004), but there was no significant difference between the different professions in total pain signs listed (KW = 10.26, *p* = 0.12). Respondents that had experienced an operation gave significantly more pain signs (5 (3, 6)) than those that had not (4 (2.5, 5); MWU = 12,511, *p* = 0.006).

### Associated levels of pain and veterinary attention needed for different conditions

Bone fracture was considered to be the most painful condition with 91.6% of respondents assigning it to be severely painful and none thinking it wasn’t painful (Table 3). It was also the condition with the highest number of respondents (98.8%) stating that veterinary attention needed to be sought the day it occurred (Table 3).

Overgrown cheek teeth, sores on hocks, bite wounds and osteoarthritis, similarly were described as painful by all respondents (Table 3). Neutering for both female and male rabbits had the highest percentages of respondents stating they were not painful (2.4% and 2.8% respectively, Table 3).

The majority of conditions were commonly thought to require veterinary attention the same day or within 2–3 days. However, there were some people responding “does not need veterinary attention” for every condition with the exception of bone fracture, overgrown back teeth, and gut stasis (Table 3).

### Level of pain scored for videos of rabbits in a clinical setting

A total of 283 respondents completed the second part of the questionnaire within the same session as the first, and a further 62 respondents completed it in a separate session, totalling 345 responses. The demographics of this subsample were similar to that of the entire sample, with the majority being female (95.4%) and most commonly aged 25–34 years (31.6%).

Respondents’ total pain score was calculated by combining the pain score of all eight videos (each scored 0–3) and ranged from 3 to 22 out of a possible 24 with a median total pain score of 12 (10, 14). This median score was lower than the expert total score of 18.

Each video was given a range of scores by respondents (Table 4). Videos three and four had the most scores the same as the expert consensus with 91.6% and 86.1% respectively (Table 4).

**Table 4:** Percentage of 345 respondents identifying pain at each level. * denotes scores corresponding to the experts’ consensus

Video number	Percentage of respondents using each pain score			
	0	1	2	3
1	9.0	32.5*	44.3	14.2
2	4.1	27.2	45.5*	23.2
3	91.6*	7.2	1.2	0
4	86.1*	13.3	0.6	0
5	26.4	24.3*	27.8	21.4
6	12.2	8.1	22.9	56.8*
7	6.7	24.3	40.9*	28.1
8	2.0	5.5	18.8	73.6*

**Fig. 1:**
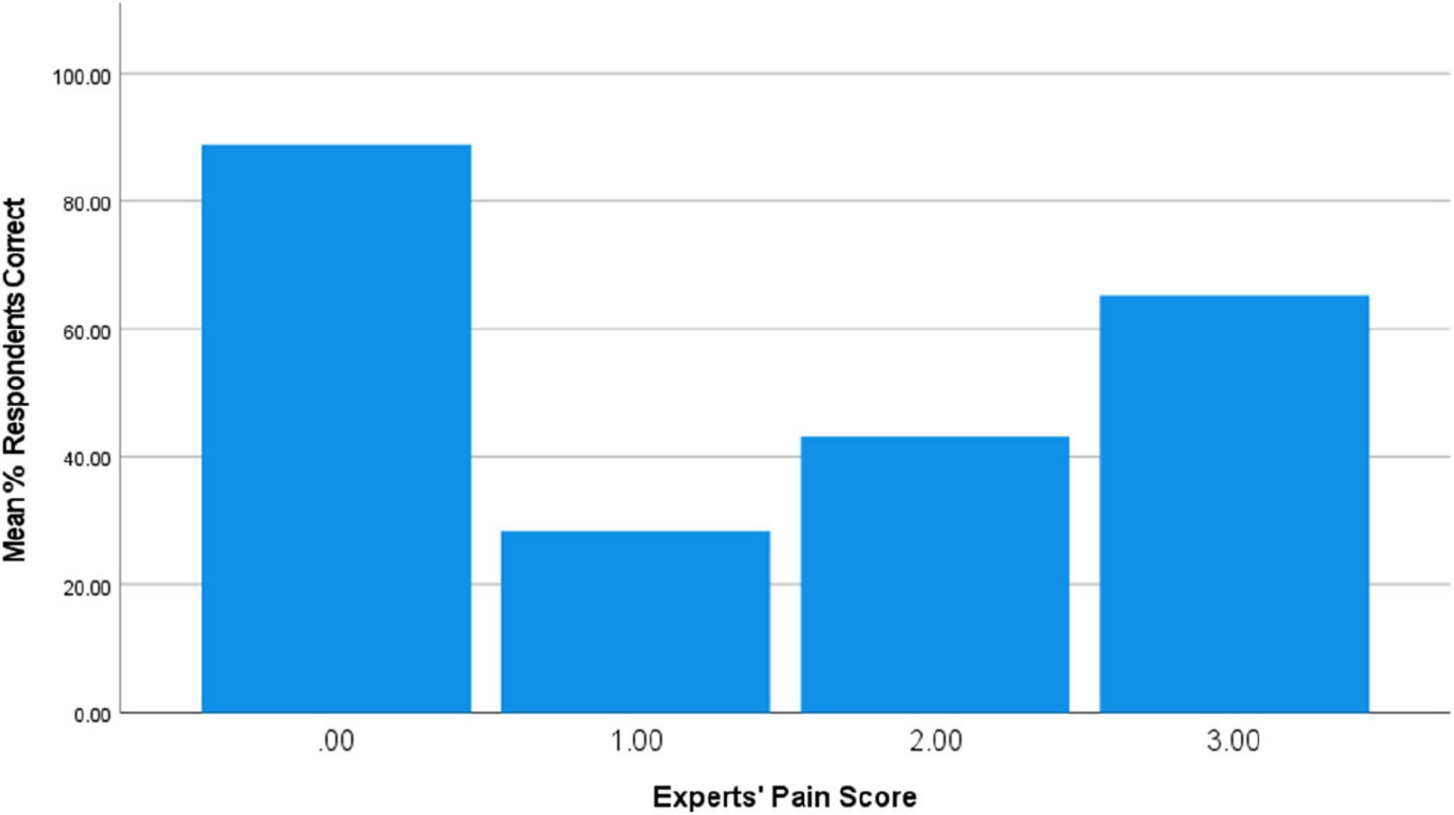
Percentage of correct responses to videos showing each pain score

The videos of rabbits with an expert score of three; severe pain, had the majority of respondents correctly identify the level of pain (video six: 56.8% and video eight: 73.6%; Table 4). In contrast, the videos of rabbits in mild and moderate pain were more often incorrectly identified (Table 4; Fig. 1).

The likelihood of a respondent stating the rabbit required veterinary attention increased with pain score (Table 5).

**Table 5:** Percentage of 345 respondents identifying pain level in agreement to the experts and stating that veterinary attention was needed

Expert pain score	% of respondents scoring correctly	% of respondents stating veterinary attention needed
0 – non painful	88.8	4.9
1 – mild pain	28.4	59.6
2 – moderate pain	43.2	79.3
3 – severe pain	65.2	87.2

The median number of correctly scored videos was 5 (4,5), only 1.4% of respondents scored all videos in agreement with the expert consensus. The deviation from expert score ranged from 0 to 13 with a median deviation of 4 (3,6).

For the videos of rabbits not in pain the majority stated that the rabbits were showing normal behaviour. Several observed that the rabbit was not eating properly or rubbing their chin or scratching their ear was the reason for their higher pain score.

For rabbits in mild pain, lack of movement and eating were frequently commented upon. Comments for rabbit 5 also included that it showed no signs of pain and some respondents were unsure as to whether this rabbit was comfortable and sleeping or in pain.

For rabbit 2 in moderate pain, the most frequently commented sign of pain was the rabbit’s posture with many respondents also noting failed grooming attempts. In contrast for rabbit 7, change to locomotion was most commonly noted.

For rabbit 8 (severe pain), most respondents commented on shaking and fewer correctly identified increased respiratory rate as the cause of the shaking. In contrast, rabbit 6 also severe pain had a high percentage of respondents comment on posture and locomotion.

### Associations between pain scoring and participant demographics

When considering total pain score, there was no significant difference between genders = (MWU = 2642.5, *p* = 0.348), nor between those that worked with rabbits and those that did not, (MWU = 8080, *p* = 0.051) or between those who had or hadn’t experienced an operation (MWU = 5976. *P* = 0.463). However, age had a significant, small, positive correlation with total pain score (Rho = 0.143, *p* = 0.008). There was also a small positive correlation between total pain score and the time for which a respondent was able to observe their rabbit (Rho = 0.128, *p* = 0.018).

Deviation from expert score did not differ significantly with gender (MWU = 1783, *p* = 0.147) or age, but those working with rabbits had slightly lower deviations, i.e. were more accurate (4 (3,5) vs 4 (3,6); (MWU = 10,946.5, *p* = 0.052). Deviation didn’t vary between current and past owners (MWU = 4450, *p* = 0.294), nor with total number of rabbits owned (Rho = -0.086, *p* = 0.112), but there was a weak negative correlation with time owning rabbits (Rho = -0.137, *p* = 0.011).Those who had experienced an operation generally had smaller deviations (4 (3, 5)) than those that had not (5 (4, 7); U = 12,511.5 *p* = 0.006).

## Discussion

This paper described the first examination of how reliably owners can assess pain in their pet rabbits. A total of 91.8% of the 500 respondents agreed with the statement that rabbits feel pain to the same extent of other animals. This level of recognition is likely beneficial for the welfare of rabbits. Interestingly 7.8% of respondents thought that rabbits feel pain more than other animals.

Owners were on average able to list five signs of pain. Since there are at least thirteen signs, there is a potential need for additional education, especially regarding the rarely reported signs such as changes in grooming behaviour and faecal changes. Anorexia was the pain sign most commonly reported by respondents (83.6%) and was considered to be the most important sign (47.4%). In their tool for quantifying acute, post-operative pain Benato et al., [13] did not use anorexia as pain indicator since it is difficult to assess within the short timescale relevant to peri-operative care. However, it is an important indicator of the general health of the animal and, potentially, a more reliable pain sign for owners at home, where they may encounter chronic pain as well as the acute pain focused on in the BRPS.

The second most common pain sign mentioned was posture (63.4%) and a large percentage of respondents mentioned other signs that are included in the BRPS as well as more specific descriptors such as “hunched” (46.4%) and “abdomen pressing” (13.0%). This sign was similarly commonly mentioned in the justifications respondents gave when pain scoring the videos. With this use of language already in some rabbit owners’ vocabulary, this would suggest that the BRPS or similar tool could easily be adapted for use by owners to further improve pain assessment at home.

A total of 4.8% of respondents mentioned the Grimace scale specifically when giving pain signs which shows some knowledge of this tool. The Grimace scale was originally developed for rabbits undergoing laboratory procedures and quantifies pain using changes in facial expression. Changes such as orbital tightening, cheek flattening, nose shape, and altered whisker and ear positions are included [7].

This Grimace scale was later combined with clinical parameters to create the multidimensional CANCRS composite scale [19], which has been validated for construct validity and inter-rater reliability. The CANCRS scale is reliable for use with multiple breeds of rabbits that may have different morphology. The Rabbit Pain Behavioural Scale (RPBS [20]) like the BRPS [13] is a new addition. These scales were not mentioned by survey respondents, which is unsurprising since they had only recently developed when the study took place.

Respondents scored the pain associated with various conditions similarly to experts reported in previous studies. Both Keown et al. [4] and Benato et al. [16] polled veterinarians and found orthopaedic surgery to be considered the most painful veterinary procedure for rabbits. Here, owners similarly reported bone fracture to cause the highest levels of pain (severely painful: 91.6%). Owners considered female neutering (severely painful: 33%) to be more painful than male neutering (severely painful: 22%) which aligns with previous studies [4, 16]. However, owners scored these procedures as less painful than did veterinary professionals [4, 16]. These two procedures also received the highest percentage of responses stating that they were not painful (female 2.4%; male 2.8%). This may be because neutering is commonplace and thus owners are desensitised to the associated pain, or because they believe the use of analgesics given to rabbits during this procedure ameliorates all pain.

Education around the emergency care needed for bone fractures, gut stasis, and flystrike would seem to be needed as although most respondents (98.8%, 96.8% and 94.8% respectively) correctly stated that these conditions need same day veterinary attention, there were still 1.2% for bone fracture, 3.2% for gut stasis and 5.2% for flystrike that were unaware. Hay poke to the eye and bite wounds had the highest percentage of participants reporting that they did not need veterinary attention (3.6% and 3.2% respectively), which may be due to owner’s experience and knowledge of treating these conditions at home. However, both conditions would also benefit from analgesia prescribed by a veterinary professional to ensure all welfare needs are met.

The majority of respondents reported learning pain signs from their own experience (78.4%), whilst veterinary advice was reported to be the second most common source (49.0%). This is surprising given that 85.2% of owners experienced their rabbit going through an operation, when we may have expected veterinarians to have shared knowledge about pain indicators. Hence, there is potential for enhanced education at this time. While there may be opportunities for veterinary professionals to impart their knowledge during routine consultations, operations and post-operative care, there is research showing that veterinarian-client communication can be challenging for both parties [21]. Even if there was better communication within this setting, only 63% of rabbits are neutered, and only 50% receive regular vaccinations [6], which limits the contact of rabbit owners with veterinary professionals to facilitate knowledge dissemination. Other sources of information such as social media and other internet sources have become increasingly popular with 56.2% of dog owners and 51.8% of cat owners reporting using Facebook groups to receive health information [22], and 33.8% of rabbit owners here reported to have learnt pain signs from social media (33.8%). When searching for the term “signs of pain in rabbits” in the commonly used search engine Google™, 8.3 million results appear which gives a very large base of sources for the average rabbit owner to access if they so desired. Much of this information lacks evidence-base and is of variable quality, making it hard for owners to know which sources to trust.

In Part two of the study, respondents scored rabbits with different levels of pain. Overall, 88.8% correctly identified the rabbits which were not in pain. This may be because most owners are familiar with seeing normal pain-free rabbit behaviour at home. The majority of respondents also correctly identified rabbits in severe pain (65.2%), but were not so good at correctly identifying mild or moderate pain; 28.4% and 43.2% of respondents correct respectively. It is likely that because the behaviour of rabbits in extreme pain is so different from pain-free rabbits, it is easier to identify than more subtle behaviour seen in rabbits that are in mild or moderate pain, which are also challenging to distinguish from one another. The concept of midpoint values being harder to distinguish is a common phenomenon (e.g. see [23]). It would be beneficial to research whether further training could improve owners’ abilities to identify mild and moderate pain. This could be done by owners scoring videos of rabbits in pain, as done here, but pre- and post-exposure to a training resource. Comparison between the accuracy of trained and a control group would help ascertain the value of training.

In-depth analysis of the respondents’ answers also suggests that targeted education on the motivations behind different rabbit behaviours would be beneficial as there were some common behaviours that were commonly misidentified or missed. For rabbit eight, while the majority of observers noted the rabbit shaking (80.9%), only 53.3% noted increased respiratory rate. There was also some misidentification of the “chinning” behaviour seen in video three. This is a normal scent marking behaviour, but several owners thought it was caused by dental pain. Although the veterinary context may have led respondents to this answer, it suggests that not all owners are familiar with all aspects of normal rabbit behaviour.

Those that worked with rabbits and those that had experienced an operation listed significantly more pain signs. Maybe those working with rabbits had more exposure to normal rabbit behaviour or had received education about different pain signs from veterinary professionals. Similarly, owners may have educated themselves to prepare for post-operative care or have gained experience witnessing pain.

When scoring the videos, the time a respondent had been a rabbit owner was weakly, negatively correlated with their deviation from expert score (*p* = 0.011), suggesting more experienced owners were more accurate. This is as predicted as they would likely have had more experience of pain and hence be better able to recognise it. Additionally, those that had experienced their rabbit undergo an operation and those that worked with rabbits, showed marginally lower deviations, suggesting greater accuracy in pain scoring. This further suggests that exposure to rabbits and in particular those in pain, as well as training can improve ability to recognise pain. Unlike past research, we saw no difference in scoring levels or accuracy with gender [24], but a significant weak positive correlation between respondent age and total pain score, suggesting that older respondents rated pain higher. These findings are similar to those reported in Benato et al. [16].

The time a respondent was able to observe their rabbit for, showed a significant weak, positive correlation with their total pain score which could be due to owners having more opportunity to observe normal rabbit behaviour and therefore being more sensitive to abnormal rabbit behaviour indicative of pain. It is also possible that owners who are able to observe their rabbits for longer do so as they are more worried about what their rabbit is doing, and this could also lead them to score pain higher, but this would require further research. This is the first survey to ask owners about how much time they would be able to see their rabbit to observe their behaviour. In this sample the majority of owners reported being able to observe their rabbit(s) for more than 6, but less than 12 h a day (25.9%). This seems high and likely reflected a potential bias in our sample as does the large number of indoor rabbits that are more likely to live in the same environment as the owner giving them opportunity to observe the rabbits longer. However, there were still 82 respondents that could see their rabbit(s) to observe them for less than 2 h a day.

The number of respondents who had cared for rabbits after neutering (78.2%) is slightly higher than the 63% of rabbits reported to be neutered in the PDSA PAW report [6], further suggesting that the population may not be representative of the general rabbit population and highlighting further potential bias. This may be due to the recruitment of participants via social media with a large proportion of those likely following the Rabbit Welfare Association and Fund. These owners are likely keener and more knowledgeable [whether through their own experience of observing their own rabbits or further research] than the majority of rabbit owners and hence while our assessments of knowledge may overestimate the general rabbit owning population, the associations affecting ability to pain score remain meaningful.

It is likely that the answers given in this survey were affected by social biases, some owners may report what they believe is socially acceptable, which may not fully reflect their own behaviour. Lack of finances commonly limit pet owners’ ability to seek veterinary services, and hence they may be reporting their ideal, rather than typical behaviour, and it’s likely that fewer owners would seek prompt veterinary care than reported it was necessary. It is also noteworthy that when filling in a survey, and watching videos about pain, the respondents may have been extra vigilant and sensitised to subtle signs of pain, which they may be less likely to spot during their usual day-to-day animal care. As a preliminary study, we have conducted a series of univariable analyses. It would now be valuable to conduct a hypothesis-driven study in which multivariable analyses explore the combination of variables which best predict an individual owner’s accuracy at, and level of pain scoring.

## Conclusion

Our results provide the first insight into how rabbit owners identify pain and their general ability to apply this knowledge to identify pain accurately. The majority of rabbit owners within this sample were able to list numerous pain signs and could generally identify rabbits not in pain and those in severe pain. However, their ability to distinguish between mild and moderate pain was limited and shows that some education and improvement in this area would be beneficial for pet rabbits in the future.

The study also highlights an apparent gap in the communication between owners and veterinary professionals. Speaking to, and informing, owners when animals are having procedures is imperative in gaining better outcomes for patients and is a well-placed opportunity to educate owners on the signs of pain in rabbits and should be capitalised on by the veterinary community.

## Methods

### Questionnaire development

A two-part questionnaire was created using JISC Online Surveys™ (Supplementary materials). Part one was completed by all respondents and included 17 questions and 49 sub-questions within three sections (A-C); mainly closed multiple choice. Section A collected demographic information including respondents’ experience with rabbits, and whether this was in a professional capacity. Section B asked about their current rabbit’s routine and to avoid bias, participants were asked to answer about the rabbit whose name came first alphabetically, or the rabbit that they last owned if they no longer owned any rabbits. The final section of Part one (Section C), aimed to understand the respondents’ thoughts and beliefs about pain in rabbits by asking them where they learnt about rabbit pain and to what extent they believed rabbits felt pain. They were also asked to name all pain signs they knew, their opinion of the most important sign of pain and give estimations of the pain level associated with 14 different conditions.

### Pain scoring of videos

Part two, Section D, was optional and could also be completed at a later date (within a month), if respondents did not have time straight away. If so, they provided an email address and were sent Part two, along with a unique identification number, so that answers could be matched. Section D required respondents to watch eight video clips of rabbits. These were selected from those filmed pre- and post-routine veterinary procedures and used during the creation of the BRPS [13].

Based on the scores on BRPS, we selected 14 videos which showed diversity in pain scores. The videos were cut from around 5 min to 30 s, which was deemed sufficient to allow an accurate assessment, whilst still maintaining respondent engagement. The three authors, one exotics specialist clinician (VB), one vet nurse (CF) and one behaviour expert (NJR), who are all experienced in observing rabbit behaviour and rating pain, scored the clips using a simplified scale. Whilst the BRP has 21 points and requires veterinary professions to rate seven different aspects of pain, this “owner scale” only included four points:

- Level 0: not in pain
- Level 1: mild pain
- Level 2: moderate pain
- Level 3: severe pain

From the initial 14 videos, we selected eight which all three authors agreed upon (two not in pain (Level 0), two in mild pain (Level 1), two in moderate pain (Level 2) and two in severe pain (Level 3). Although the original videos included each rabbit pre- and post-surgery, we selected only one video per rabbit to ensure independence of scoring. The order in which the videos were presented was randomised for pain score, but all respondents saw them in the same order.

Respondents were asked to watch each video clip in full, then to score it on the four-point scale. In order to test the ability of owners to use the simple scale, no further training or additional descriptors were given. Respondents also reported whether they thought the level of pain would require veterinary intervention. Finally, they were asked to describe why they gave the chosen pain score. In total there were 73 sub-questions in Part 2, if respondents completed all parts (Supplementary Materials 1). The software enabled respondents to review and edit responses ahead of submitting, but all questions were mandatory, so no incomplete responses were received.

### Subject recruitment

The primary questionnaire was open from 16th of February 2022 through to the 7th of March 2022. Those that opted to complete the additional Section D at a later date, had until 16th March 2022 and were emailed reminders ten and five days before the deadline.

The study was promoted through social media using its own Facebook page created by the author (CF) for this purpose and via the Rabbit Welfare Association and Fund (RWAF), various rabbit rescues and veterinary practices and posted in UK rabbit owner groups (Supplementary Materials 2). To attract a diverse sample, a prize draw incentive was utilised. Respondents had the chance to win one of ten Burgess Pet Care rabbit goody bags and those who completed the optional second part were offered an additional entry into the draw. Respondents had to be over 16 years of age and to currently, or previously, have owned a rabbit.

### Data handling

The survey responses were described descriptively and were also used to create ten quantitative variables (Table 6).

**Table 6:** Variables extracted from questionnaire Sect. 1 and optional Sect. 2 and used in quantitative analysis

Section	Variable	Definition
1	Age	Age of participant in years
	Gender	Gender of participant (male, female or non-conforming)
	Total number of rabbits owned	Total number of rabbits owned currently and previously
	Work with rabbits	Whether the participant was currently working with rabbits (0/1)
	Time been rabbit owner	How long the participant had been a rabbit owner (in years)
	Experience of operations	Whether the participant had experienced an operation with one or more of their rabbits (0/1)
	Total number of pain signs	Total number of pain signs listed by the participant
2	Total pain score	Sum of pain scores participant gave to all eight videos (out of 32)
	Deviation from expert score	Sum of difference between participant score and the expert score, summed for all eight videos

The answers to the open question that asked respondents to name pain signs were used to create the variable of ‘total number of pain signs’. Answers were examined to see if they mentioned each of the six BRPS categories [13]. Key words and phrases were searched for each pain sign (Table 7). If any of the key words or phrases for a category were mentioned, the respondent was given a score of one. Any other signs given by more than 5% of the population were also counted. These scores were then added together to give a further variable: the total number of BRPS pain signs out of a maximum score of twelve. Terms such as ‘hunched’, ‘abdomen pressing’ and ‘lethargy’ were noted, but not counted as separate categories since they are descriptors used in the BRPS, so were counted within the relevant BRPS category. Participants were also asked where they learnt this information.

**Table 7:** Key words and phrases used by respondents and classified into each separate pain sign category

Category	Key words and phrases used by respondents
Demeanour	quiet, dull, lethargy, subdued, interest, responsive
Posture	lying, laying, sitting, pushing, pressing, hunched, abdomen pressing
Locomotion	movement, move, active, inactivity, still, lethargy
Eye position	eye, squinting, tightness, closing
Ear position	ear, back, flat
Grooming	grooming, groom, dirty
Anorexia	eating, not eating, not interested in food/treats, in appetent, inappetence, lack of appetite
Teeth grinding	tooth, teeth, grinding
Grimace scale	Grimace scale
Noises	noise, squealing, screaming, grunting, groaning, moaning
Faecal changes	faeces, poo, poop, toileting
Physiological	breathing, respiratory, heart rate, temperature

The most important pain signs listed by respondents were similarly classified using the same categories and an additional category for those that stated that all pain signs were of equal importance. When respondents listed more than one sign, only the first sign listed was used, unless they specifically stated that one was more important than the other. We similarly classified the reasons why respondents gave their chosen pain score for each video. However, comments related to the video quality, clarity, and length, as well as comments that suggested that the respondent had taken cues from the environment to allocate pain level, were also classified.

In Section D, a total pain score given was created by adding together all eight of the pain scores a respondent reported. We then compared the scores given to the expert scores, to derive the total deviation from expert score. This was calculated by subtracting the expert pain score from the respondent’s pain score for each video, and the absolute differences were summed to derive a total absolute deviation irrespective of whether scores were higher or lower than the expert score.

### Statistical analysis

Statistical analysis was performed using SPSS 26 (IBMM). Data were not normally distributed, hence we used non-parametric tests throughout. Mann–Whitney U tests were used when comparing two independent samples and Kruskal Wallis tests for three or more. Spearman’s correlation allowed comparison of two ordinal or continuous variables.

We explored whether the total number of pain signs listed in Part 1 varied with gender or age of respondent, whether they worked with rabbits and whether they had experienced an operation. We similarly compared whether the total pain score and the accuracy of pain score (measured as deviation from experts) when scoring the videos in Part 2, varied with the same factors, and with how long owners could see their rabbits in an average day. The two continuous dependent variables from Part 2 (total deviation from expert and total pain score) were tested and found to explain less than 50% of the variation in one another. Therefore, each dependent variable was tested separately against the relevant independent variables. *P* < 0.05 was considered statistically significant.

### Supplementary Information

**Supplementary Material 1.**

**Supplementary Material 2.**

## Data Availability

The datasets used and analysed during the current study are available from the corresponding author on reasonable request.
