# Supplementary material for: An investigation into how accurately UK rabbit owners identify pain in their pet rabbits
Source: BMC Vet Res. 2024 Mar 27;20:122. doi: 10.1186/s12917-024-03947-7 (PMC10967183; doi:10.1186/s12917-024-03947-7)
Supplement: Supplementary file 1 — Supplementary Material 1. [file 12917_2024_3947_MOESM1_ESM.docx]

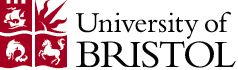


Thank you for taking the time to complete this questionnaire.

The questionnaire has **three** sections for you to complete:

**Section A** is all about you and your experience with rabbits. Please note that no personal details that could identify you will be requested.

**Section B** is about your rabbit’s routine. You will be asked to answer about only ONE rabbit that you currently own or if you no longer own rabbits the last one that you owned.

**Section C** is to find out your thoughts around pain in rabbits.

There are no more than 7 questions in each section, totalling 18 questions, which should only take less than 10 minutes. If you have any questions before participating in this study additional information can be found on the participant information sheet linked here.

Please confirm that you:

- Currently have or previously owned a rabbit
- Are over the age of 16
- Have read the above information and consent for my data to be collected and used for the purposes stated above

SECTION A

1. How old are you?

- 16-24
- 25-34
- 35-44
- 45-54
- 55-64
- 65-74
- 75+

1. Which gender do you identify with the most?

- Male
- Female
- Gender variant/non-conforming
- Not listed
- Prefer not to say

1. (i) Do you currently or have you ever worked within an animal-related profession working with rabbits?
   1. No
   2. Yes

3. (ii) If you answered yes to the previous question in what profession is/was this?

a. vet

b. vet nurse

c. animal carer

d. work in pet outlet e.g. pet shop

e. working in rehoming

f. other. Please specify ……

1. In total, how long have you been a rabbit owner?
   1. Less than 6 months
   2. 6 months to a year
   3. 1-5 years
   4. 6-10 years
   5. 11-15 years
   6. More than 15 years
2. How many rabbits do you currently own?
   1. 0
   2. 1
   3. 2
   4. 3
   5. 4
   6. 5+
3. How many rabbits have you owned previously excluding the rabbits you currently have?
   1. 0
   2. 1
   3. 2
   4. 3
   5. 4
   6. 5+
4. If you currently do not own a rabbit how long ago did you last own a rabbit?
   1. In years
5. i) Have any of your rabbits had an operation whilst in your care?
   1. Yes
   2. No

7. ii) If so, which type of operation have your rabbit(s) had? Please select all that apply

a. Neutering (castration in males or spay in females)

b. Orthopaedic (bone repair)

c. Foreign body removal (removal of object swallowed)

d. Removal of lump or cyst

e. Dental (e.g. teeth trimming or teeth removal)

g. Other. Please specify…………………

Section B Your Rabbit’s Routine

**If you currently have more than one rabbit, please select the rabbit who’s name comes first alphabetically and answer the following questions about them. If you don’t currently have rabbits, please answer about the last rabbit you owned**

1. What is the name of the rabbit you will be answering these questions about?

…….. ………………………………………………………………

1. Who do/did you consider to be his/her main caregiver?
   1. Yourself
   2. A child/ children within the household
   3. Another adult within the household
   4. Both you and a child/children within the household
   5. Both you and another adult within the household
   6. Other (please specify)
2. Is/was your rabbit’s main living space (please tick one)
   1. □ A hutch or cage in the house without attached run
   2. □ A hutch or cage in the shed , garage or outbuilding  without attached run
   3. □ A hutch or cage in the garden without attached run
   4. □ A hutch or cage in the house with an attached run
   5. □ A hutch or cage in the shed , garage or outbuilding  with an attached run
   6. □ A hutch or cage in the garden with an attached run
   7. □ A shed or other outbuilding in which it can roam freely
   8. □ It’s a house rabbit with access to one or more rooms
   9. □ Other (please specify)?………………………………………………………................
3. For how long in the average day would you be able to see what your rabbit is doing?
   1. Less than 10 minutes
   2. 10-29 minutes
   3. 30 minutes to 2 hours
   4. More than 2 hours to less than 6 hours
   5. More than 6 hours to less than 12
   6. 12 hours or more
4. How often do/did you do the following activities with your rabbit?

|  | Never | Less often than monthly | Monthly | Every 2 weeks | Weekly | Several times a week | Daily |
| --- | --- | --- | --- | --- | --- | --- | --- |
| Check weight |  |  |  |  |  |  |  |
| Brush/groom |  |  |  |  |  |  |  |
| Clip nails |  |  |  |  |  |  |  |
| Check cleanliness of bottom |  |  |  |  |  |  |  |
| Check ears |  |  |  |  |  |  |  |
| Check front teeth |  |  |  |  |  |  |  |

Section C – Understanding Pain

1. Please select the statement you agree with the most
   1. Rabbits feel pain to the same extent as other animals such as dogs
   2. Rabbits feel pain but less than other animals such as dogs
   3. Rabbits feel pain more than other animals such as dogs
   4. I am unsure if rabbits feel pain
   5. I do not think that rabbits can feel pain
2. What signs do you look for that would show if your rabbit(s) are in pain? Please list as many signs as you can think of.
3. Of the signs you have given above, which do you think is the most important sign to you to spot if your rabbit is in pain?
4. Where did you learn about the pain signs you stated above? (select all that apply)
   1. Online websites
   2. Book
   3. Veterinary advice
   4. Friends and Family
   5. Social media groups
   6. Own experience
   7. Other…. Please specify……………………….
5. How painful do you think the following conditions are for a rabbit?

|  | Not painful | Somewhat painful | Painful | Extremely painful |
| --- | --- | --- | --- | --- |
| Bone fracture |  |  |  |  |
| Overgrown back teeth |  |  |  |  |
| Gut stasis (gut stops working and low faecal output) |  |  |  |  |
| Fly strike (dirty and maggot infested bottom or surrounding area) |  |  |  |  |
| Red, inflamed hocks (reddened areas or bleeding of underside of heels or feet) |  |  |  |  |
| Ear infection |  |  |  |  |
| Urine scalding (persistent contact of urine on the skin causing sores and wounds) |  |  |  |  |
| Female rabbit being neutered (spay) |  |  |  |  |
| Male rabbit being neutered (castration) |  |  |  |  |
| Haypoke (sharp hay or other object scratching the eye) |  |  |  |  |
| Thorn in foot |  |  |  |  |
| Bite wound |  |  |  |  |
| Osteoarthritis (stiff joints) |  |  |  |  |
| Lump or swollen area |  |  |  |  |

1. If you were to see any evidence of the following symptoms and conditions, how quickly do you think you would need to seek veterinary advice?

|  | Does not need veterinary attention | After 14 days | Within 8-14 days | Within 4-7 days | Within 2-3 days | Same day |
| --- | --- | --- | --- | --- | --- | --- |
| Bone fracture |  |  |  |  |  |  |
| Overgrown back teeth |  |  |  |  |  |  |
| Gut stasis (gut stops working and low faecal output) |  |  |  |  |  |  |
| Flystrike (Dirty and maggot infested bottom or surrounding area) |  |  |  |  |  |  |
| Red, inflamed hocks (reddened areas or bleeding of underside of heels or feet) |  |  |  |  |  |  |
| Ear infection |  |  |  |  |  |  |
| Urine scalding (persistent contact of urine on the skin causing sores and wounds) |  |  |  |  |  |  |
| Haypoke (sharp hay or other object scratching the eye) |  |  |  |  |  |  |
| Thorn in foot |  |  |  |  |  |  |
| Bite wound |  |  |  |  |  |  |
| Osteoarthritis (stiff joints) |  |  |  |  |  |  |
| Lump or swollen area |  |  |  |  |  |  |

NEW PAGE

Thank you for completing this survey!

Would you be willing to answer a few more questions?

This will take around 15 minutes and includes watching 8 video clips of rabbits and answering as to whether you think they are in pain or not. It will allow you to have another chance in the prize draw.

1. Would you be able to answer a few more questions?
   1. Yes
   2. Yes but I don’t have the time at the moment
   3. No

(If don’t have time at the moment)

Please could you give us an email address to send the additional questions to for you to complete at a time convenient to you. ……………………………………………….

Additional questions (if answer yes)

You will be asked to watch 8 videos of 30 seconds and then asked 3 questions about each video. Please watch each video to completion before answering the questions.

Please note that all rabbits were under veterinary care for routine procedures and were treated for any pain they may exhibit. Some rabbits may have catheters in their ears as indicated by red bandaging, which is routine in any animal when hospitalised and allows administration of medication and fluids.

1. Rabbit 1 - Please watch this short video.
   1. What level of pain would you score this rabbit to be in out of 3 with 0 being no pain and 3 being severe painful?
      1. 0 – no pain
      2. 1 – mild pain
      3. 2 – moderate pain
      4. 3 – severe pain
   2. Why have you selected this score?
   3. Would this level of pain indicate to you that this rabbit needed vet intervention?
      1. Yes
      2. No
2. Rabbit 2 - Please watch this short video.
   1. What level of pain would you score this rabbit to be in out of 3 with 0 being no pain and 3 being severe painful?
      1. 0 – no pain
      2. 1 – mild pain
      3. 2 – moderate pain
      4. 3 – severe pain
   2. Why have you selected this score?
   3. Would this level of pain indicate to you that this rabbit needed vet intervention?
      1. Yes
      2. No
3. Rabbit 3 - Please watch this short video.
   1. What level of pain would you score this rabbit to be in out of 3 with 0 being no pain and 3 being severe painful?
      1. 0 – no pain
      2. 1 – mild pain
      3. 2 – moderate pain
      4. 3 – severe pain
   2. Why have you selected this score?
   3. Would this level of pain indicate to you that this rabbit needed vet intervention?
      1. Yes
      2. No
4. Rabbit 4 - Please watch this short video.
   1. What level of pain would you score this rabbit to be in out of 3 with 0 being no pain and 3 being severe painful?
      1. 0 – no pain
      2. 1 – mild pain
      3. 2 – moderate pain
      4. 3 – severe pain
   2. Why have you selected this score?
   3. Would this level of pain indicate to you that this rabbit needed vet intervention?
      1. Yes
      2. No
5. Rabbit 5 - Please watch this short video.
   1. What level of pain would you score this rabbit to be in out of 3 with 0 being no pain and 3 being severe painful?
      1. 0 – no pain
      2. 1 – mild pain
      3. 2 – moderate pain
      4. 3 – severe pain
   2. Why have you selected this score?
   3. Would this level of pain indicate to you that this rabbit needed vet intervention?
      1. Yes
      2. No
6. Rabbit 6 - Please watch this short video.
   1. What level of pain would you score this rabbit to be in out of 3 with 0 being no pain and 3 being severe painful?
      1. 0 – no pain
      2. 1 – mild pain
      3. 2 – moderate pain
      4. 3 – severe pain
   2. Why have you selected this score?
   3. Would this level of pain indicate to you that this rabbit needed vet intervention?
      1. Yes
      2. No
7. Rabbit 7 - Please watch this short video.
   1. What level of pain would you score this rabbit to be in out of 3 with 0 being no pain and 3 being severe painful?
      1. 0 – no pain
      2. 1 – mild pain
      3. 2 – moderate pain
      4. 3 – severe pain
   2. Why have you selected this score?
   3. Would this level of pain indicate to you that this rabbit needed vet intervention?
      1. Yes
      2. No
8. Rabbit 8 - Please watch this short video.
   1. What level of pain would you score this rabbit to be in out of 3 with 0 being no pain and 3 being severe painful?
      1. 0 – no pain
      2. 1 – mild pain
      3. 2 – moderate pain
      4. 3 – severe pain
   2. Why have you selected this score?
   3. Would this level of pain indicate to you that this rabbit needed vet intervention?
      1. Yes
      2. No

(For both) Before you go… Would you like to entered into the prize draw? Would you like to be notified of the results?

Please let us know below

- I would like to be entered into the prize draw for a chance to win one of 5 x Burgess prizes
- I would like to be notified of the results of this survey

Please provide your email-address below so you may be contacted. This will be stored securely and separately to any other data

…………………………………………………………………………………………………………………………………………………………..

Thank you for taking the time to complete this survey.
