# Supplementary material for: An investigation into how accurately UK rabbit owners identify pain in their pet rabbits
Source: BMC Vet Res. 2024 Mar 27;20:122. doi: 10.1186/s12917-024-03947-7 (PMC10967183; doi:10.1186/s12917-024-03947-7)

**Supplementary information - recruitment information**


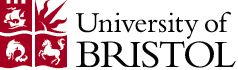


Do you own a rabbit? Or have you owned one in the past? Please can you help by sparing a few minutes to complete this survey about pain in rabbits. As a final year Veterinary Nursing student at the University of Bristol, I am undertaking research into pain in pet rabbits. The questionnaire is designed to provide insight into how rabbit owners notice pain in their pets and help us better protect rabbits in the future.

All participants will be given the chance to win one of 10x Burgess Excel Goody Bags. You also have the option to take part in a second longer phase which will give an additional two entries into the prize draw.

<https://svs.onlinesurveys.ac.uk/rabbitowners_and_pain>

Thank you for taking the time to complete the questionnaire and helping us to improve rabbit welfare. For any queries or questions regarding this project I can be contacted by email [qi18500@bristol.ac.uk](mailto:qi18500@bristol.ac.uk)


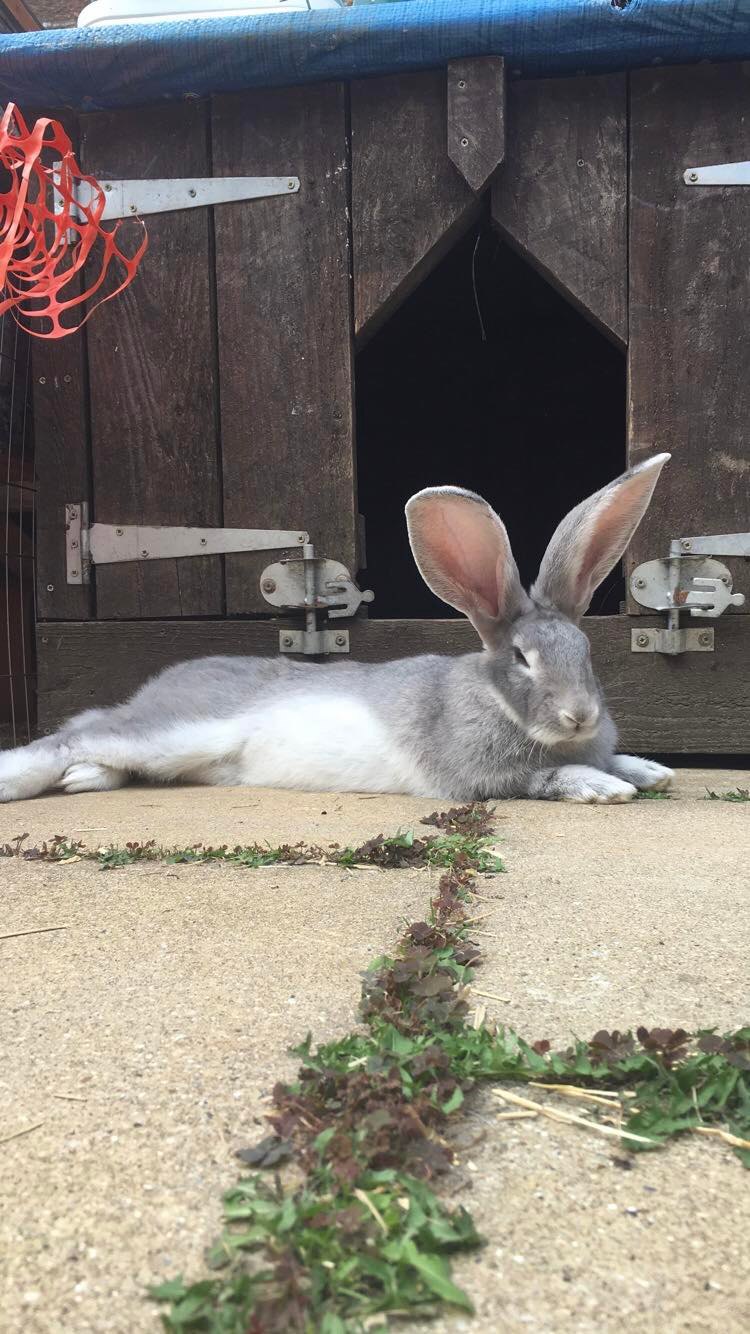

Supplement: Supplementary file 2 — Supplementary Material 2. [file 12917_2024_3947_MOESM2_ESM.docx]
